# Odorant Receptors Signaling Instructs the Development and Plasticity of the Glomerular Map

**DOI:** 10.1155/2015/975367

**Published:** 2015-01-20

**Authors:** Pablo Valle-Leija

**Affiliations:** Department of Physiology, McGill University, Montreal, QC, Canada H3G 1Y6

## Abstract

The olfactory system provides a great opportunity to explore the mechanisms that underlie the formation and function of neural circuits because of the simplicity of its structure. Olfactory sensory neurons (OSNs) located in the peripheral olfactory epithelium (OE) take part in the initial formation and function of glomeruli in the olfactory bulb (OB) inside the central nervous system. Glomeruli are key in the process of transduction of olfactory information, as they constitute a map in the OB that sorts the different types of odorant inputs. This odorant categorization allows proper olfactory perception, and it is achieved through the anatomical organization and function of the different glomerular circuits. Once formed, glomeruli keep the capacity to undergo diverse plasticity processes, which is unique among the different neural circuits of the central nervous system. In this context, through the expression and function of the odorant receptors (ORs), OSNs perform two of the most important roles in the olfactory system: transducing odorant information to the nervous system and initiating the development of the glomerular map to organize olfactory information. This review addresses essential information that has emerged in recent years about the molecular basis of these processes.

## 1. Introduction

**Figure 1:**
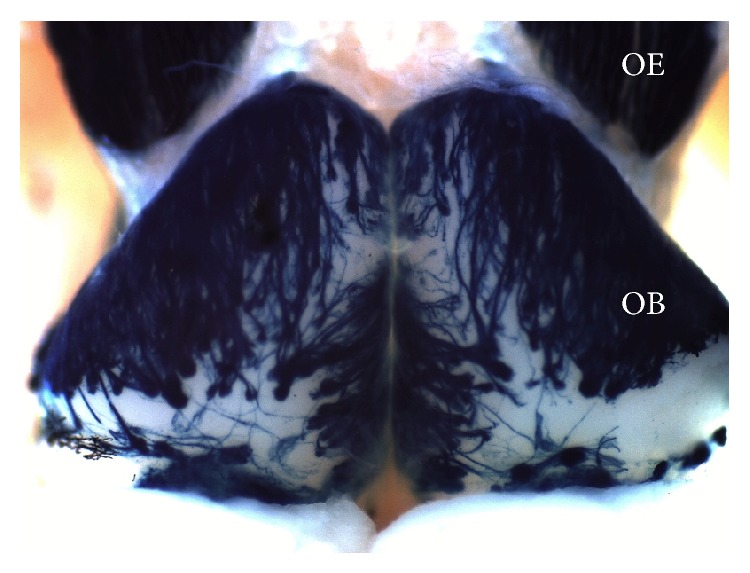
The glomerular map in the OB. OSNs in the OE project their axons to form glomeruli on the surface of the OB. The dorsal view of the OB of an OMP- (olfactory marker protein-) tau-LacZ mouse is shown. Beta-galactosidase (blue) activity was revealed by X-gal.

Each OSN population in the OE expresses only one type of OR and projects to a fixed location in the OB to form glomeruli [1, 2] which together form an anatomical map (Figure 1) activated stereotypically depending on the odorant type [3, 4]. This specific activation brings about the perceptual- and behavioral-inducing properties of odorants [5–7]. The OSNs not only face the task of directly recognizing odorants and transducing the chemical signal into an electrical signal but also actively participate in the formation and remodeling of the glomerular map during development and adult life [2, 6, 8–10].

The present review addresses the molecular basis that allows OSNs to transduce olfactory information and the different factors that determine the formation, position, and plasticity of specific glomeruli in the OB, which together form the glomerular map (Figure 1).

## 2. Anatomical Organization and Olfactory Transduction: From the OE to the OB

Two main structures comprise the mammalian olfactory system: the OE, where the OSNs reside, and the OB, where the sets of OSN axons form glomerular structures. These two structures alone codify olfactory perception in just 140 ms [11]. Glomeruli intervene in this fast coding, with each one containing up to 10,000 axons from OSNs and dendritic trees of less than 20 mitral neurons [12, 13]. Glomeruli, along with mitral and tufted neurons, process information as separate modules, in a similar way to cortical columns [14]. OSNs express a particular OR coalesce in at least two olfactory glomeruli, located in the lateral side of the OB (next to the eye socket) and medial side (toward the other OB), resulting in two similar glomerular maps in each OB [1, 15, 16], giving a minimum of four clusters of glomeruli to process the information of a single OR. However, there are a few OSN populations that converge in only one glomerulus in each OB, close to the midline where the lateral and medial map bind [17]. Finally, and most importantly, the positions of the different glomeruli are highly conserved and stereotyped between individuals, if not exactly, in a completely enclosed area of about 30 glomeruli [10, 17].

### 2.1. The Role of ORs in the Coarse Anatomical Organization of the OE and OB

To better understand the crucial role of OSNs in the anatomofunctional organization of the glomerular map, I will describe the ORs in detail. Under the premise that ORs interact with G proteins, Richard Axel and Linda Buck identified a large family of genes expressed in OSNs [18]. Now we know that this family of genes code for ORs that begin the process of olfactory transduction and perception. ORs typically contain seven transmembrane domains coupled to trimeric G proteins [18]. Currently, the most reliable accounts indicate that the OE of mice contains 1300 different ORs, each one encoded by a single gene in the mouse genome. Humans, with less olfactory sensitivity than rodents, have approximately 500 genes encoding ORs, although 40% of them are considered to be pseudogenes [19, 20]. Interestingly, each OSN expresses just one type of OR, exclusive of the other types, monoallelically [21] due to the existence of a negative feedback mechanism that depends on the OR itself [22–24].

**Figure 2:**
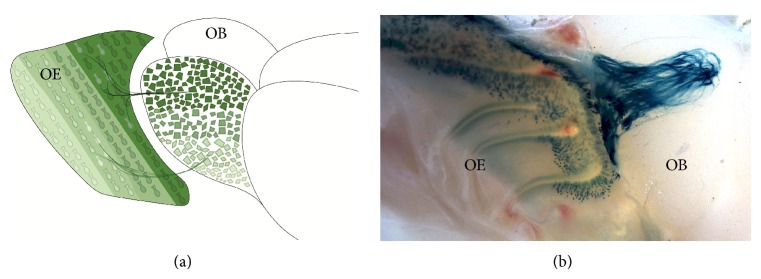
Regionalization of OSN populations in the OE and the corresponding position of glomeruli in the OB. (a) There is a gradational regionalization of the different OSN populations across the dorsoposterior-ventroanterior axis. A dorsoventral correspondence of glomerular position holds up in the OB. (b) The lateral view of the OB of a M72-tau-LacZ mouse is shown. M72 neurons are located in the dorsoposterior OE and the M72 glomerulus in the dorsal part of the OB. Beta-galactosidase (blue) activity was revealed by X-gal.

OSN populations form specific glomeruli in very stereotyped positions in the OB [1], but is there any organization for the different OSN populations in the OE? One discovered so far is the gradual regionalization of the expression of ORs along the dorsoposterior-ventroanterior axis of the OE [25–27]. Each of these OSN populations forms, corresponding to the expression zone, glomeruli across the dorsoventral axis of the OB (Figure 2(a); [28]). Thus, for example, the M72 OSN population located in the dorsoposterior part of the OE targets the dorsal part of the OB (Figure 2(b)). The differential expression of transcription factors and axon guidance molecules in the OSNs underlies this organization [29, 30].

### 2.2. OR Activation, Odorant Signal Transduction to Glomeruli, and the Odortopic Map

**Figure 3:**
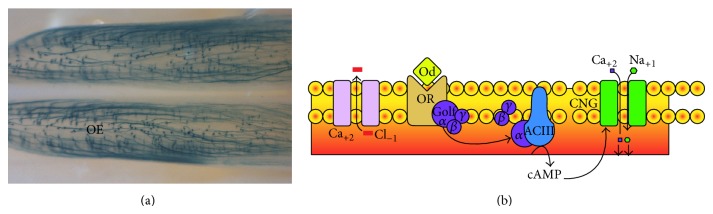
OSNs in the OE and OR signaling pathways. (a) The OSN somas and cilia (dots), where the OR signaling pathways occur, transduce the information to the axons (lines) through action potentials that eventually reach the OB. (b) Diagram of the signaling pathway of a standard OR after activation. The interaction of odorants with their receptors on the OSN cilia and soma leads to Golf activation, which activates ACIII, thus causing increased cAMP levels. Next, cAMP activates CNG channels that allow the entry of sodium and calcium. Finally, this latter ion activates a chloride channel that contributes to depolarization enough to reach the threshold for action potentials. Modified from [38].

The beginning of the transduction of olfactory information to the nervous system occurs when the volatile molecules interact with the ORs located in the OE (Figure 3(a)). The OR transmembrane segments TM3, TM5, and TM6 seem to be responsible for this interaction, and together they form a binding pocket responsible for the affinity of the odorant for its receptor [31, 32]. Golf protein activation in OSNs which follows odorant binding transduces the chemical signal into electrical. This chain of events stimulates adenylate cyclase (AC) type III to produce cAMP (Figure 3(b); [33, 34]) activating the effector cationic CNG channel permeable to potassium, sodium, and calcium. This latter ion is important in two ways: it increases the depolarization of the OSN and it also induces the opening of a chloride channel that allows chloride outlet (Figure 3(b)) enough to reach action potentials [35, 36]. The CNG channel in the OSNs is a tetramer comprised of two subunits of the CNGA2 protein and two accessory units CNGA4 and CNGB1b. In knockouts of the CNGA2 subunit, the channel cannot be formed and the electrical response induced by odorants is completely abolished [37].

One OR recognizes multiple odorants, and one odorant can activate multiple ORs [39]. Therefore the question immediately arises of what would be the activation code for each odorant? The answer is that, because the information of each OR converges in specific glomeruli within the OB, each odorant selectively activates a particular set of glomeruli, thereby forming a stereotypic map that gives the odorant a particular identity. In this respect, several studies have shown that, at least with stimuli of a single molecule, a particular set of glomeruli is activated, and this response is conserved between individuals [3, 4, 40, 41]. However, the activation pattern evoked from complex odors, formed by several molecules, is not the result of the sum of glomeruli activated by each molecule [40]; there are competitive odorant interactions, among other factors, that do not result in a summation [39, 42–44].

OSNs expressing similar ORs form glomeruli in surrounding regions and respond to similar odorants [45], which is why the activation patterns involve several neighboring glomeruli. Regions of the OB which have been identified respond primarily to aldehydes, ketones, alcohols, and so forth, and therefore, the anatomical glomerular map underlies the formation of the odortopic map. In other words, particular odorants are encoded in specific glomerular regions of the OB [28, 46]. However, these activated glomeruli are distributed throughout the OB due to the fact that different chemical groups of the odorant molecule activate several ORs that are different in sequence [28].

## 3. Formation, Position, and Plasticity of Specific Glomerular Circuits

One of the most interesting questions about the olfactory system is how the glomerular map, constituted by all glomeruli, is formed and remodeled. To answer this, we must identify the molecular factors that participate in the formation and position of specific glomeruli in the OB. In the past decade, it was shown that the OE is formed from the olfactory placodes located in the anterolateral region of the head. These oval structures appear by embryonic day 9 and bring about the entire population of OSNs. By embryonic day 11, we can observe what will be the OB in the most frontal region of the nervous system [47]. In the 16-17th days, OSN axons penetrate the external layer of the OB, before arrival of the periglomerular interneurons and mitral-tufted dendrites [48, 49], and from this point the formation of glomerular structures is induced [10, 15, 50]. Thus, It is important to emphasize that the formation of glomeruli during development is not determined in the OB itself, since the axons of the OSNs induce the formation of these, so we should not say that OSN axons converge but instead coalesce and form glomeruli [49]. Most of the glomeruli are well established and structured at an early postnatal stage [10, 49–51], before the organization of the associated circuitry [48, 49].

### 3.1. Visualizing Specific Glomerular Circuits

**Figure 4:**
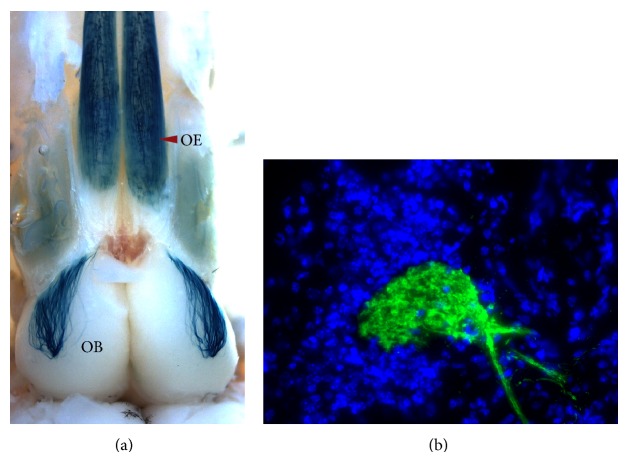
OSN in the OE that expresses the same type of OR projects axons to specific coordinates in the OB forming glomeruli. (a) The OB and the OE of a M72tLacZ mouse are shown (dorsal view). Neurons and axons that contain beta-galactosidase activity (blue) are revealed by X-gal. The M72 neurons, scattered throughout the OE, project their axons to the dorsal-posterior part of the OB and form at least one glomerulus. (b) OB coronal slice (20 *μ*m) of a M72tGFP mouse. The M72 glomerus, surrounded by periglomerular cells (stained with DAPI, blue), only contains axons (green) from the M72 OSN population.

One of the most important technical advances in regard to the formation and position of glomeruli was the development of genetically modified animals that allow observation of only one glomerular circuit (Figure 4; [1]). In these mice, the endogenous allele of a particular OR gene was modified by adding, after the transcription termination, a site for ribosome entry (IRES, internal ribosome entry site), which serves as another translation start point, which together with the green fluorescent protein (GFP) or beta-galactosidase coupled to the tau protein allows the visualization of axonal projections (Figure 4(a); [1]). Thus the formation of the glomerular map was studied with greater precision [1, 15]. In this regard it was observed that the formation of anterior glomeruli begins prenatally [10, 50] and posterior glomeruli form after birth [15]; mature glomeruli contain axons from only one OSN population (Figure 4(b); [2]); in postnatal day 5 there are at least two glomeruli per OB, which can be drastically remodeled during the first 2 weeks after birth [2, 10, 50] and to a lesser degree in adulthood [8].

### 3.2. The Role of the ORs, Their Signaling Cascade, and Axon Guidance Molecules in the Position and Formation of Specific Glomeruli: Coarse Axonal Targeting and Local Positioning

One of the first hypotheses proposed that glomerular development was mediated by OR homophilic interactions between axons [52–55]. This rather simple hypothesis assumed that the OR found in the growth cones and axon terminals of OSNs [56, 57] attaches or bonds the axons of the same OR/OSN population. Experiments in which particular amino acids of the specific ORs M50, P2, and M72 are modified produce new glomeruli located close to the glomerulus that corresponds to the wild-type sequence [53, 54]; also homophilic interactions between cells expressing the same ORs have been proven to exist* in vitro* [55].

However, despite some evidence in favor of the OR homophilic interaction hypothesis, there is strong evidence that proves beyond doubt that the homophilic interactions are not at all necessary to explain axon targeting, fasciculation, glomerular formation, and position in the OB [58–60]. The studies we previously referred to [53, 54] were conducted with a small number of OR genes and few nucleotide modifications. Furthermore, they were not followed by exhaustive modification of the OR sequence with the corresponding correlation of glomerular position. In addition, it was determined that the position of the OR gene in the genome is also critical to define the position of the corresponding glomeruli in the OB. When an OR gene is replaced by another, new glomeruli form in ectopic places, but near enough to the stereotypic position that corresponded to the deleted OR [54, 61]. Therefore, the indirect influence that the OR gene expression exerts on the position of its glomeruli is contextual or relative to its position in the genome, and thereby chromatin regulation of axon guidance genes and transcription factors might be different.

The sequence of the OR, its position in the genome, and the regionalization of the OSN population are critical factors that influence the location of the glomeruli in the OB, but the very existence of the glomeruli depends on the OR signaling cascade. In experiments where the I7 OR is mutated to prevent its coupling with G proteins, the OSNs that express the mutant receptor are unable to form glomerular structures [58], and when constitutive G proteins are expressed in the I7 circuit, the formation is reestablished. It has been suggested that the pathway by which this process proceeds is through the Gs (s-stimulatory) proteins also present in OSNs [62]. Interestingly, the first G protein to appear during development is the Gs, followed by Golf. Thus, it has been proven that Gs proteins regulate coarse axonal targeting to the olfactory bulb and Golf local axonal segregation [60].

Previously, we mentioned that the region of expression of the OR in the OE influences the dorsoventral (D-V) position of glomeruli in the OB. But what factors could influence the anterior-posterior (A-P) position? One important difference between the A-P positioning in relation to the D-V is that the A-P is not regulated by the anatomical location of OSNs in the OE but by OR-derived cAMP signals [60]. Interestingly, when different mutants of Gs proteins with different levels of activity are expressed in the I7 population, a change in the position of the I7 glomerulus is manifested in the anterior-posterior axis. The least active G protein (Gs) formed I7 glomeruli in more anterior regions and the one with the highest activity in posterior regions [58]. Thus, the intracellular levels of cAMP influence the developmental formation and the position along the anterior-posterior axis of specific glomeruli in the OB [32, 58], independently of odorant induced activity [60].

The position and formation of glomeruli are regulated differentially by the OR signaling cascade. First, ACIII deletion disrupts the formation of anterior glomeruli and locally changes the position of posterior glomeruli in the OB. Thus, for example, in the absence of ACIII, P2 axons cannot form glomeruli, but M71 axons can, although the local segregation is minorly affected. Nonetheless, if the coding sequence of the M71 receptor is replaced (→) with the sequence of the P2 OR gene, M71→P2 axons, instead of forming the normal dorsal-posterior M71 glomerulus, they project to the ventral-posterior position without forming any glomeruli [33, 34]. In contrast, when the olfactory effector channel CNG is eliminated, the coalescence of M72 axons is altered and P2 axons remain almost the same as the wild type [37], and so it can be suggested that electrical activity and OR signaling have a different role for anterior and posterior OSN populations. One important factor that might explain this difference is that ORs cloned from the anterior OB produce lower levels of agonist-independent OR signaling than the ones from the posterior OB. Also, axon targeting molecules (e.g., Neuropilin-1 and Plexin-A1) and glomerular segregation molecules (e.g., Kirrel 2 and Kirrel 3) are differentially expressed along the anterior-posterior axis and have different susceptibilities to agonist-dependent and agonist-independent OR activity [60].

Hitoshi Sakano's team has shown that expression of various adhesion and signaling molecules is directly dependent on the following factors: the type of OR, its position in the genome, the OR signaling pathway, and the electrical activity of the OSNs [59, 60, 63], which together determine the position and formation of glomeruli in the OB [53, 63, 64]. After an exhaustive analysis of gene expression in OSNs, Serizawa et al. showed that adhesion molecules Kirrel 2 and Kirrel 3 are expressed in an inverse manner on neighboring glomeruli. Kirrel 3 molecules have adhesive interactions between themselves, but not with Kirrel 2 molecules, and* vice versa*. The MOR28 population has low expression of Kirrel 2 and high expression of Kirrel 3, and thus their axons expressing Kirrel 3 attract each other. In contrast, the MOR256-17 axons have low expression of Kirrel 3 and high expression of Kirrel 2. Repulsive interactions between axons from neighboring glomeruli are regulated in a similar way through the inverse expression of the Eph-A5 receptor and its ligand ephrin-A, thus increasing the degree of specificity of axonal coalescence in the OB. This inverse molecular expression that produces specific adhesive and repulsive interactions between axons was observed in other OSN populations and was consistent in most of them [63].

In summary, the type of OR and its position in the genome are very important in determining the location of its glomeruli in the OB and are achieved through the agonist-independent expression of axon guidance molecules [54, 60]. The structural formation of any glomerulus depends on the typical signaling cascade of ORs and the local expression of axon segregation (adhesive and repulsive) molecules [58, 60, 63]. All these factors combined can explain the formation of thousands of glomeruli, each one containing axons from only one OSN population. However, we need to know which rules are applicable to which OSN populations.

### 3.3. The Role of Spontaneous Electrical Activity in the Formation of Specific Glomeruli

The electrical activity of the OSNs has a primary role in the formation of the olfactory map [64]. This electrical activity can be divided into two types: spontaneous electrical activity and electrical activity induced by odorants. Both types have been studied in the context of formation and plasticity of the glomerular circuit. The consensus in the field is that spontaneous electrical activity is critical during the initial formation of specific glomeruli [64], whereas sensory activity does not greatly influence this initial formation, but rather the refinement, maturation, and subsequent remodeling [2, 8, 9, 65, 66]. The molecular mechanisms that underlie the influence of spontaneous activity on glomerular formation relate to the differential expression of adhesive and repulsive molecules in the OSN axons [63].

Spontaneous electrical activity can be divided into two types: spontaneous action potentials and spontaneous synaptic potentials. Both types have been modified in two ways to study glomerular formation, first, in only one population of OSN and, second, in all populations: through overexpression of potassium channel Kir 2.1, it was concluded that the disruption of spontaneous neuronal firing of the P2 population altered the formation of P2 glomeruli, with even a total absence of formation [64]; and through the expression of tetanus toxin, which blocks synaptic vesicle release, in the P2 population causes the absence of P2 axon coalescence and formation of glomeruli. However, in both cases, if altered in all OSN populations, the disruption of glomerular formation is significantly attenuated, though differentially, in some OSN populations more than others. Interestingly, the conditional disruption of spontaneous neural firing of a particular OSN population causes the loss of the corresponding glomeruli in adult mice [64]. Additionally, the loss of glomeruli and OSN populations can also depend on the activity evoked by odorants in a competitive environment [67]. In conclusion, the different OSN populations have to compete with each other to form and maintain their glomeruli, which is compatible with the theory of Darwinian neural development [68].

### 3.4. Olfactory Stimulation Has Different Effects on the Formation and Plasticity of Specific Glomeruli

The glomerular circuit has become a great model to study sensory-dependent plasticity due to the great advantage of visualizing specific OSN populations and glomeruli and having one odorant molecule that preferentially activates them [39, 69, 70]. In a controlled olfactory environment, sensory experience is not required for the initial and coarse formation of the glomerular map, mainly because it does not influence the basal electrical activity and the expression of axon guidance and adhesion molecules [2, 41, 63, 64, 71]; nonetheless, specific olfactory stimuli can produce other changes in the fine features of the glomerular map and circuitry. Here, we summarize a few examples: (1) an increase in M71 glomerular size in response to Pavlovian conditioning with acetophenone [8]; (2) the presence of M71 and M72 supernumerary glomeruli in adulthood caused by olfactory deprivation during the early postnatal stage [2]; (3) the acceleration of the process of refinement of I7 glomeruli by conditioned exposure to odorants [66]; (4) increased survival of periglomerular cells of glomeruli activated by specific odorants in a Pavlovian conditioning paradigm [72]. In summary, different olfactory stimulation patterns can influence the plasticity of the glomerular map.

**Figure 5:**
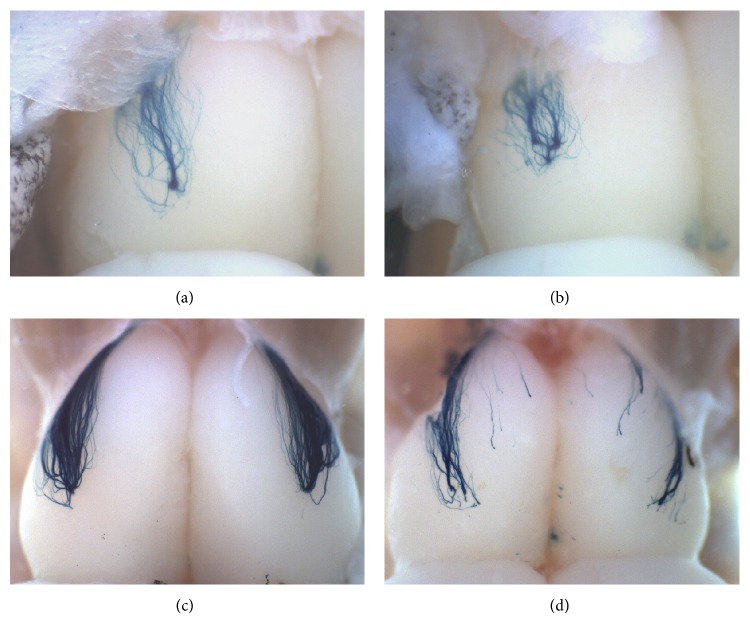
Chronic exposure to pure acetophenone influences the formation of M72tLacZ glomeruli in the OB. (a) A lateral M72 glomerulus in one OB of a 20-day-old M72tLacZ mouse. Typically, only one lateral glomerulus is formed in all mice. (b) Formation of M72 supernumerary glomeruli after 20 days of acetophenone exposure from birth. (c) Regeneration of lateral M72 glomeruli in both OBs in a M72tLacZ adult mouse 45 days after methimazole treatment. (d) Dispersed and mistargeted M72 axons with no glomerular formation in an adult mouse exposed to acetophenone for 35 days after methimazole treatment (days 10–45).

These examples of sensory-dependent plasticity produce minor changes. Nonetheless, one phenomenon in which odorants can drastically influence the formation of the glomerular map is chronic exposure to highly concentrated odorants, which causes the formation of more glomeruli if the exposure is made during the early postnatal stage [9]. This was studied using M72tLacZ (Figures 5(a) and 5(b)) and I7tGFP knock-in mice; it was shown that the effect is circuit-specific; in other words, acetophenone only affects the M72 circuit and heptaldehyde only affects the I7 circuit, both of which are specific ligands of these ORs. These two circuits have different degrees of susceptibility to the specific ligand [9]. Along this line, the effect of odorant exposure during the regeneration of OSNs in the adult stage has been explored. Methimazole induces the degeneration of the OE and OSNs in the first few days after the injection [10]. It has been proven that, in a control olfactory environment, the number of M72 OSNs almost is reestablished and the formation and position of M72 glomeruli are very similar to control mice (Figure 5(c); [10]). Likewise in another study it was shown that there is a recovery of the functional topography of odor representations in the OB after OE degeneration [73]. However, under chronic exposure to pure acetophenone for 35 days (from day 15 to day 45 after methimazole treatment), M72 glomeruli cannot be formed again; also there is an incorrect regional targeting of M72 axons, like the incursion into the anterior-medial region (Figure 5(d)). The anterior-posterior targeting of axons is odorant-independent during development [60] but might change in adults under these experimental conditions.

Chronic odorant exposure changes OSN axonal segregation in the OB during development [60], which may be the underlying cause for the formation of supernumerary interconnected M72 glomeruli caused by acetophenone (Figure 5(b); [9]) and also might relate to the absence of M72 glomeruli in the adults treated with methimazole and exposed to acetophenone (Figure 5(d)). These experiments have the future goal of studying whether these phenomena have an impact on the odortopic activation of the OB and whether it can change innate olfactory responses through preference/aversion tests or more subtle differences testing odorant-detection thresholds and discrimination between different types of odorants and enantiomers. Finally, we want to investigate whether these structural changes in glomeruli (Figures 5(b) and 5(d)) can be inherited transgenerationally, something which has been proven for M71 glomeruli increased size caused by Pavlovian conditioning using acetophenone [74].

## 4. Perspectives and Conclusions

I emphasize the fact that we still do not know all the factors that participate in the formation of the glomerular map. The different OR/OSN populations behave in very different ways, which makes it hard to find common rules that can be adapted to a general model. What we know is a part of the answer, which is mainly related to the activity of specific OR/OSN populations and their associated signaling machinery, in addition to the important role of electrical activity in glomerular formation. I think it is important to find the precise molecular mechanisms by which the different OSN populations segregate their axons and form specific glomeruli in a stereotypical position in the OB and to know if the differences in the mechanisms of the diverse OSN populations follow a common logic. Our current perspective may well be correct but not generalizable, and more studies are needed that repeat the same findings in other OR/OSN populations. It will also be important to analyze systematically and globally the relationship between the expression of axon guidance molecules, the position of glomeruli, and the signaling pathway of the different ORs. It is essential to note that not much is known about the maturation process of OSNs and the further axonal integration into a particular glomerular circuit during adulthood. Up until now, the experiments have focused mainly on the formation of glomeruli during development without paying much attention to the maintenance process of the glomerular map that is carried out due to the continuous neurogenesis in the OE. Future studies that can effectively analyze the time course of maturation of the OSNs will be important for an overview of the process and to unify the dataset obtained so far. Finally, in this context, it is critical to determine whether the anatomical organization of the glomerular map is crucial for the perceptual properties of each and every odorant and how the odortopic identity is maintained after the OB.
